# What Encourages Physically Inactive People to Start Running? An Analysis of Motivations to Participate in Parkrun and City Trail in Poland

**DOI:** 10.3389/fpubh.2020.581017

**Published:** 2020-11-17

**Authors:** Ewa Malchrowicz-Mośko, Patxi León-Guereño, Miguel Angel Tapia-Serrano, Pedro Antonio Sánchez-Miguel, Zbigniew Waśkiewicz

**Affiliations:** ^1^Institute of Sport Sciences, Eugeniusz Piasecki Academy of Physical Education, Poznań, Poland; ^2^Health, Physical Activity and Sports Science Laboratory, Department of Physical Activity and Sports, Faculty of Psychology and Education, University of Deusto, Bilbao, Spain; ^3^Department of Didactics of Musical, Plastic and Body Expression, Faculty of Teaching Training, University of Extremadura, Badajoz, Spain; ^4^Department of Sports Medicine and Medical Rehabilitation of I.M, Sechenov First Moscow State Medical University, Moscow, Russia; ^5^Institute of Sport Sciences, Jerzy Kukuczka Academy of Physical Education, Katowice, Poland

**Keywords:** motivation, marathon runners, running, beginner runners, lifestyle

## Abstract

The aim of this study was to investigate the motivations for beginner runners to take part in Parkrun Poznań and City Trail Poznań, Poland, taking into account their socio-demographical variables. A total of 165 (age: 36.33 ± 10.38) inexperienced runners participated in the diagnostic survey and completed the MOMS (Motivations of Marathoner Scale) questionnaire. The sample consisted of 82 men (49.7%) and 83 women (50.3%). The results showed that men were more likely to start running due to competition-related motivations, while the motivations of women were more often related to affiliation, psychological coping, life meaning, and self-esteem. As age increased, the level of motivation due to personal goal achievement, competition, and recognition scales decreased. The Affiliation Scale was especially important for singles who started running, in comparison with runners who were married or in a relationship. These factors should be taken into consideration by event managers and public health specialists. Promoting safe running among people who have no experience with this sport is as important as encouraging them to run. All runners stated that they would like to run a marathon in the future. Moreover, thanks to initiatives such as City Trail and Parkrun, Polish respondents feel motivated to lead an active lifestyle, with an average score of 4.98 on a 7-point Likert scale.

## Introduction

There is worldwide concern about insufficient levels of physical activity among a large part of the population. Physical inactivity is the fourth leading risk factor for global mortality and has been estimated to cause 6% of deaths worldwide. Physical activity can reduce the risk of cardiovascular disease, overweight, obesity, falls, type 2 diabetes, stress, and depression. The most important benefits of regular physical activity include lower prevalence of many diseases, as well as a decrease in mortality. People of all ages can enjoy the numerous physical, psychological, emotional, and social benefits that physical activity brings (1–4). An inactive lifestyle is associated with higher all-cause mortality, coronary artery disease, hypertension and stroke. It is also a primary cause of most chronic diseases, as the body rapidly adapts to insufficient physical activity which, if continued, results in substantially reduced quantity and quality of life. Regular physical activity can significantly improve mental health, self-confidence, healthy aging, and quality of life (5). Increasing levels of physical activity to meet current guidelines during adulthood is a public health priority. Researchers, sports events organizers and health promotion professionals are exploring the reasons why some people are physically active, whereas others are not (6). Motivation to participate in sport is highly complex, and is a key factor that influences individuals' initiation and maintenance of active behavior. Motivation affects sport participation and is a critical factor in exercise adherence.

Parkrun is an initiative that organizes free, weekly 5 km timed runs (every Saturday morning) (7). It has grown on an international scale and promotes sport for everyone, regardless of their running experience, results, age, gender or social status. It has the potential to increase physical activity and promote health, especially among social groups with low economic status (8). Parkrun runs are held on almost all continents and bring together hundreds of thousands of amateur runners. Over the last few years, this initiative has grown from a small-time trial in Bushy Park, London, to a global social movement. Parkrun is non-profit and is based on the involvement of sponsors. Organizers (usually volunteers and local community) strive to ensure that every city where there is a demand for it has the opportunity to engage in regular runs. Participation in Parkrun provides an inclusive leisure space for casual sociability, as well as facilitating a shared experience of exercising with others—especially with inexperienced runners (9).

Another example of social imitative behavior in the field of mass sport is City Trail Poland. City Trail is an initiative that was started in Poland in 2010. It was a response to a shortage of 5 km runs, in contrast to the growing number of marathons and half marathons. It is based on the assumption that runs are for everyone, including beginner runners and families with children. A beginner runner was defined as “an individual having had no prior running training and not being involved in regular sporting activities”(10). Runs are organized on a regular basis in the autumn and winter seasons in major Polish cities, and they attract up to 20,000 participants annually.

Variables such as age, gender, and educational level influence physical activity motivation, so it is important to take them into account when developing strategies to promote sport for all. The motivations of beginner and inexperienced runners have not yet been analyzed. The publications on motivation for running do not cover studies on this population group, apart from a number of analyses of the relationship between the motivation for long-distance running and socio-demographic characteristics, e.g., gender, age or place of residence. Ogles et al. (11) reported that the most important reasons (for female runners more than for male runners) were weight concern, self-esteem, affiliation, psychological coping and life meaning (12). Similarly, in the Polish context, Waśkiewicz et al. (13) found that men were more motivated by competition whereas womens' motivation was more linked to weight concern, affiliation, psychological coping, life meaning, and self-esteem. More recently, León-Guereño et al. (14), reported that Polish female athletes were more motivated by psychological coping while and men were by competition and personal goal achievement. Nikolaidis et al. (10) found that women who ran marathons were frequently focused on personal goal achievement. According to Summers et al. (15) women showed higher levels of addiction to running marathons than men (15). Malchrowicz-Mośko and Poczta (16) added that women were more likely to run than men because they wanted to escape everyday life and due to the prevailing fashion. Netz and Raviv (17) pointed out that the age of athletes was a very important factor in determining motivations for physical activity. Nikolaidis et al. (18) studied age-related aspects of marathon participation and identified that competing with other runners was the most important aspect for the youngest athletes. Malchrowicz et al. (19) found that young runners were especially focused on sports results, while older people were more centered on social interaction with other participants in mass running events such as half-marathons. At the same time, children and adolescents' motivation for participating in mass running events was associated with their fun and enjoyment rather than social motives (20).

Previous studies have analyzed the motivations of experienced runners, but we still know little about what motivates beginners– e.g., Parkrun and City Trail participants. Understanding the reasons why people decide to engage in physical activity is extremely important from the point of view of health prevention, in order to effectively promote mass sport and healthy lifestyles and encourage people to participate in sporting events. There is little research on the motivation to start the adventure of running. “Judaism? Islam? Israel's new religion is marathon running” is an example of the kind of statements found in many articles today (21). However, before the decision to participate in a marathon is made, there needs to be a previous initial impetus to take up running, e.g., during Parkrun or City Trail events. Sometimes running becomes a means of fighting cultural restrictions, as is the case of women who have limited participation in mass runs, such as the first international marathon in Tehran—TehRun (21). It is important to understand the motives of runners with wide-ranging running and socio-cultural experience to encourage a greater number of people to take up this sport (22). In the Western world, middle- and upper-class individuals are the primary participants in distance running at the non-elite level (23). According to Stempel (24), the upper classes in the United States use sports such as running to create barriers between them and the lower classes. Care for one's body, health, and physical condition are distinctive features of the ideology of healthism in Western societies, which in some people's view, makes it possible to distinguish the more physically active middle classes from supposedly inactive and lazy lower classes (25, 26). In line with the principles of healthism, it is mainly members of the middle-class who run in Poland (27). However, in the existing publications about the social class determinants of sport, there are few analyses about running (only 22 have been identified). This is probably because running escapes simple divisions into elite and non-elite disciplines, as it is a multidimensional contemporary social phenomenon. Wilson (28) study reported that more affluent people engage in sport more often than members of the lower socioeconomic classes. Parkrun or City Trail are accessible for everyone, regardless of socioeconomic class. Runners with a high social status will probably increase their distance of choice to participate in ultramarathons in the future, will travel to take part in popular or prestigious marathons, or will change the discipline they engage in, for example, by shifting to triathlon (29). It may be rather difficult to encourage some people to do physical exercise at all, let alone engaging in regular physical activity, such as persuading some members of the lower socioeconomic classes to take up running. That is why initiatives that promote mass sport on an open and accessible basis for everyone (such as Parkrun and City Trail) are so important. Given their contribution to better health status, they can have an indirect impact on lowering public health care costs.

The aim of this study was to investigate the motivations of beginner runners through MOMS scales' 9 motivational dimensions, in particular, of participants in Parkrun Poznań and City Trail Poznań, taking their socio-demographical variables into account. Within these variables, apart from previously analyzed sex, age, and education level, the influence of the family context was analyzed. As little research has been done on this variable (14). to the intention is to find out the motivational aspects that lead beginners to start running, since this participation will result in an improvement in health. When talking about motivational processes within an sport context, the type of motivation that has been related with commitment and the beginning of an activity is the intrinsic motivation (30, 31), which is a concept that comes from self-determination theory (32, 33). It is associated with behaviors promoted by the pleasure and satisfaction derived from individual willingness to participate in an activity/sport, which leads to positive consequences such as psychological welfare, interest, enjoyment, and intention to persist (32, 34). Studying motivation of Parkrun participants is important because the initiative is aimed at everyone, including members of the lower socioeconomic classes. A particularly interesting issue is also that Parkrun meetings provide opportunities for social affiliation. Taking part in them may involve a strong sense of community with other beginner runners. City Trail is the only Polish initiative entailing regular runs for beginner runners, and Parkrun is the only international running initiative for novices adopted in Poland to date.

## Materials and Methods

### Participants and Study Design

This is a descriptive, quantitative, cross sectional research; whose sample consisted of 165 Parkrun and City Trail participants, with a total of 82 men (49.7%) and 83 women (50.3%). The average age was 37 years (36.33 ± 10.38). Forty runners (24.2%) were younger than 30 years old; 66 runners (40%) were aged between 31 and 40; 45 runners (27.3%) were aged between 41 and 50; and 14 runners (8.5%) were over the age of 50. All of them provided written informed consent to participate in the research, and participants were treated ethically under the American Psychological Association ethics code.

Forty five participants (27.3%) started running at the instigation of other people (family, friends), and as many as 120 people (72.7%) made their own decision to start running. Eighty six people ran with family/friends (52.1%), and 79 people (47.9%) ran alone. All participants stated that they would like to run a marathon in the future.

A total of 105 people (63.6%) had a higher education level, whereas 60 people (36.4%) had a secondary education level. While 143 of the participants were professionally active (86.7%), 18 people were students (10.9%), 2 people were retired (1.2%), and 2 people were unemployed (1.2%).

Ninety one people (55.2%) had children and 74 people (44.8%) had no children. Whereas, 126 participants (76.4%) were married or in a relationship, 39 (23.6%) were single.

### Measurements

#### Sociodemographic Status

Following previous studies Molina-García et al. (35), participants were asked about sex (male, female), age, motivation for leading an active lifestyle, education level (secondary education or higher education), own decision to participate vs. persuasion of other people (family, friends, etc.), people with children vs. people without children (yes or no), and married status (single, married, and divorced).

#### Motivations of Marathoners'

The multidimensional MOMS scale (36), developed initially by Masters et al. (37) was used. Athletes' motivation was measured via 56 items or reasons for participating in a marathon, organized using a 7-point Likert-scale, with the highest score being 7 “very important reason,” and the least valued motive rated 1 “not a reason.” This scale shows 9 dimensions that the authors divided into four main broader groups of motives: (1) psychological motives, involving self-esteem (items: 11, 23, 29, 31, 32, 34, 53, 56), e.g., “To improve my self-esteem,” psychological coping (items: 10, 15, 18, 28, 36, 38, 39, 47, 50), e.g., “To become less anxious,” and life meaning (items:; 13, 20, 25, 27, 41, 49, 55), e.g., “To add a sense of meaning to life.” (2) Achievement-related motives, including personal goal achievement (items: 5, 9, 22, 35, 46, 51), e.g., “To improve my running speed” and competition (items: 2, 40, 43, 52), e.g., “To compete with others”; (3) social motives, showing recognition (items: 3, 6, 19, 45, 48, 54), e.g., “To earn the respect of peers” and affiliation motives (items: 7, 12, 16, 24, 30, 33), e.g., “To socialize with other runners”; and (4) physical health motives, including general health orientation (items: 8, 14, 17, 26, 37, 44), e.g., “To improve my health” and weight concern (items: 1, 4, 21, 42), e.g., “To help control my weight.” (37).

### Procedure

A diagnostic survey method was used, including a standardized interviewing technique (the research instrument developed was an online interview questionnaire). The organizers of City Trail Poznań and Parkrun Poznań consented to conducting the study in March 2020. The research was carried out in accordance with the Declaration of Helsinki, and the study was treated in accordance with the guidelines of the Publication Manual of the American Psychological Association regarding consent and anonymity. As online surveys or questionnaires do not require the completion of a separate participant information sheet or consent form, participation in the survey was deemed to constitute informed consent. Participants were informed about the significance of the study and were kindly requested to provide information. The survey was voluntary, anonymous, and confidential. In Poland, anonymous diagnostic surveys do not require approval by a bioethics committee. The survey was forwarded to City Trail and Parkrun participants by the events' organizers. The survey was created using Google Docs technology. People who had not previously engaged in running and had not led an active lifestyle according to the World Health Organization prior to participating in Parkrun and City Trail were asked to take part in the survey. This sample selection allowed us to study people who took up physical activity and running thanks to initiatives such as Parkrun and City Trail. Participants were informed that, according to the World Health Organization (38), meeting physical activity recommendations involves doing exercise for at least 150 min a week (moderate-intensity effort) or for at least 75 min a week (high-intensity effort).

### Data Analysis

The normality of distributions was assessed with a Shapiro-Wilk test and homogeneity of variance was checked using Levene's test. Intergroup comparisons were made using Student's *t-*test for independent variables or (in the case of failure to meet the assumption of homogeneity of variance) using the Cochran-Cox test. Cohen's d was calculated to determine effect size for mean comparisons. Correlations between age expressed in years and motivation scale values were performed using Pearson's linear correlation coefficient. The probability values were considered significant at *p* < 0.05. Calculations were made in Statistical 10.0.

## Results

Table 1 showed the participants' motivations. The highest-rated motivations for running were related to health orientation (5.15) and personal goal achievement (4.97), and the lowest-rated motivations were recognition (2.41) and competition (3.18).

**Table 1:** Participants' motivations (on a 7-point Likert scale).

**Motives**	**Total**		**Women**		**Men**		***t***	***p***	***d***
	***M***	***SD***	***M***	***SD***	***M***	***SD***			
Health orientation	5.15 ± 1.37		5.21 ± 1.40		5.08 ± 1.35		−0.63	0.528	0.10
Weight concern	4.08 ± 1.87		4.03 ± 1.83		4.13 ± 1.92		0.35	0.730	0.05
Personal goal achievement	4.97 ± 1.55		4.81 ± 1.52		5.13 ± 1.58		1.33	0.186	0.21
Competition	3.18 ± 1.76		2.74 ± 1.54		3.64 ± 1.86		3.38	0.001	0.53
Recognition	2.41 ± 1.35		2.35 ± 1.34		2.46 ± 1.37		0.51	0.609	0.08
Affiliation	3.79 ± 1.92		4.13 ± 1.99		3.45 ± 1.79		−2.29	0.023	0.36
Psychological coping	4.26 ± 1.61		4.79 ± 1.47		3.73 ± 1.56		−4.47	0.000	0.70
Life meaning	3.77 ± 1.56		4.18 ± 1.43		3.35 ± 1.59		−3.54	0.001	0.55
Self-esteem	4.39 ± 1.55		4.76 ± 1.37		4.02 ± 1.64		−3.12	0.002	0.49

Furthermore, Table 1 revealed that the motivations of novice runners differed by gender. Men were more likely to start running due to Competition-related motivations, whereas women were more often inclined to do so due to aspects related to Affiliation, Psychological Coping, Life Meaning, and Self-esteem. Health and Weight orientation and Personal goal achievement held similar importance for women and men.

Table 2 showed the motivations of people who made an independent decision to start running and those that did so encouraged by other people. A statistically significant difference was found on the Affiliation scale, as higher significance was seen among people who had been encouraged to run by other people (*p* = 0.000).

**Table 2:** Own decision to participate vs. persuasion of other people (family, friends, etc.).

**Motives**	**Persuaded by other people (*****n*** **=** **45)**		**Own decision (*****n*** **=** **120)**		***t***	***p***	***d***
	***M***	***SD***	***M***	***SD***			
Health orientation	5.00	1.14	5.20	1.45	−0.81	0.419	0.15
Weight concern	3.77	1.67	4.20	1.93	−1.43	0.157	0.24
Personal goal achievement	5.10	1.52	4.92	1.57	0.65	0.514	0.11
Competition	3.24	1.73	3.16	1.78	0.24	0.810	0.04
Recognition	2.73	1.36	2.28	1.33	1.88	0.062	0.33
Affiliation	4.65	1.74	3.47	1.89	3.66	**0.000**	0.65
Psychological Coping	4.16	1.43	4.31	1.67	−0.53	0.594	0.10
Life Meaning	3.63	1.38	3.81	1.63	−0.66	0.513	0.12
Self- esteem	4.42	1.37	4.38	1.62	0.15	0.883	0.02

*Notes: Bold values indicate statistically significant differences*.

The next step involved checking how motivations were shaped based on respondents' educational level (Table 3). The Life Meaning Scale was more important to people with secondary education (*p* = 0.029).

**Table 3:** People with higher education vs. people with secondary education.

**Motives**	**Secondary education (*****n*** **=** **60)**		**Higher education (*****n*** **=** **105)**		***t***	***p***	***d***
	***M***	***SD***	***M***	***SD***			
Health orientation	5.16	1.47	5.13	1.32	0.13	0.897	0.02
Weight concern	3.83	1.78	4.23	1.91	−1.30	0.195	0.21
Personal goal achievement	4.78	1.70	5.08	1.46	−1.19	0.236	0.19
Competition	3.12	1.76	3.22	1.77	−0.35	0.725	0.06
Recognition	2.45	1.49	2.38	1.27	0.32	0.748	0.05
Affiliation	4.16	1.84	3.58	1.94	1.89	0.061	0.31
Psychological coping	4.49	1.62	4.13	1.59	1.38	0.169	0.22
Life meaning	4.12	1.60	3.56	1.51	2.21	**0.029**	0.35
Self-esteem	4.44	1.61	4.36	1.52	0.31	0.759	0.05

*Notes: Bold values indicate statistically significant differences*.

It was then decided to check how participants' motivations were shaped depending on whether they had children or not (Table 4). The Recognition scale was of higher importance for people who did not have children (*p* = 0.048).

**Table 4:** People with children vs. people without children.

**Motives**	**Do you have children? Yes (*****n*** **=** **91)**		**Do you have children? No (*****n*** **=** **74)**		***t***	***p***	***d***
	***M***	***SD***	***M***	***SD***			
Health orientation	5.18	1.32	5.10	1.45	0.37	0.711	0.06
Weight concern	4.23	1.83	3.91	1.91	1.08	0.281	0.17
Personal goal achievement	4.85	1.54	5.12	1.56	−1.11	0.269	0.17
Competition	2.96	1.68	3.47	1.83	−1.87	0.064	0.29
Recognition	2.22	1.23	2.64	1.46	−1.99	**0.048**	0.31
Affiliation	3.62	1.85	4.00	2.00	−1.29	0.198	0.20
Psychological Coping	4.24	1.61	4.29	1.61	−0.20	0.840	0.03
Life Meaning	3.71	1.52	3.83	1.62	−0.51	0.612	0.08
Self- esteem	4.19	1.52	4.64	1.56	−1.84	0.068	0.29

*Notes: Bold values indicate statistically significant differences*.

An analysis was then carried out to see if the motivations were shaped depending on whether the runners were single or not (Table 5). The Affiliation scale was more important for singles who started running (*p* = 0.048).

**Table 5:** Single people vs. married people/people in a relationship.

**Motives**	**Married/in a relationship (*****n*** **=** **126)**		**Single (*****n*** **=** **39)**		***t***	***p***	***d***
	***M***	***SD***	***M***	***SD***			
Health orientation	5.24	1.38	4.83	1.33	1.65	0.100	0.31
Weight concern	4.12	1.87	3.97	1.89	0.42	0.678	0.08
Personal goal achievement	4.98	1.51	4.94	1.70	0.12	0.904	0.02
Competition	3.14	1.75	3.31	1.82	−0.52	0.601	−0.09
Recognition	2.32	1.31	2.67	1.46	−1.41	0.160	−0.25
Affiliation	3.63	1.94	4.32	1.78	−1.99	**0.048**	−0.37
Psychological Coping	4.18	1.64	4.54	1.46	−1.23	0.219	−0.23
Life Meaning	3.69	1.58	4.01	1.51	−1.12	0.262	−0.21
Self- esteem	4.33	1.57	4.60	1.50	−0.97	0.335	−0.18

*Notes: Bold values indicate statistically significant differences*.

Pearson's *r* correlation was used to check whether motivations differed by age (Table 6). Statistically significant correlations were obtained for Personal goal achievement (*r* = −0.16; *p* = 0.040), Competition (*r* = −0.18; *p* = 0.021), and Recognition (*r* = −0.21; *p* = 0.006).

**Table 6:** Scale correlations with age.

**MOMS scale**	***r***	***p***
Health orientation	0.15	0.062
Weight concern	0.04	0.579
Personal goal achievement	−0.16	0.040
Competition	−0.18	0.021
Recognition	−0.21	0.006
Affiliation	−0.06	0.440
Psychological Coping	−0.03	0.669
Life Meaning	−0.08	0.328
Self- esteem	−0.15	0.063

These were weak, negative correlations (*r* < 0.30). As age increased, the motivation values on the scales decreased.

Respondents were also asked whether Parkrun and City Trail had encouraged them to lead an active lifestyle (Table 7).

**Table 7:** Motivation for leading an active lifestyle.

	**Total**		**Items motivation for leading an active lifestyle in accordance with WHO recommendations**													
			**1**		**2**		**3**		**4**		**5**		**6**		**7**	
	***M***	***SD***	***n***	***%***	***n***	***%***	***n***	***%***	***n***	***%***	***n***	***%***	***n***	***%***	***n***	***%***
Motivation for leading an active lifestyle in accordance with WHO recommendations	4.99	1.72	10	6.06	5	3.03	18	10.91	22	13.33	36	21.82	37	22.42	37	22.42

## Discussion

The aim of this study was to analyze the reasons that lead beginner athletes to take part in Parkrun and City Trail Poznań, taking into account their socio-demographical variables. Previous studies on Parkrun have focused on its potential benefits and its impact on runners' physical and mental health and well-being. Assessments of the impact that participation in Parkrun meetings had on runners' overall level of physical activity found an increase in activity after 6 months, but this effect became less visible after 12 months (39). The increase was most pronounced among people with low levels of physical activity, which became close to the recommended weekly level due to their participation in Parkrun (40). Although participation in Parkrun did not cause that the weekly level of activity of all participants to reach the recommended levels, it is worth considering that that even low physical activity levels bring significant physical and mental health benefits (41, 42). As for the impact of Parkrun runs on the participants' weight, qualitative and quantitative studies have shown that Parkrun participants reported a decrease in weight; for example, overweight people noticed a weight loss of nearly 2.5% after 1 year without controlling their diet (43). The results of our study on Parkrun and City Trail participants in Poznań City who had not previously run and did not lead an active lifestyle showed that these initiatives encouraged them to lead an active lifestyle in accordance with WHO guidelines on an average level of 4.98 points on a 7-point Likert scale. This is a positive result from the point of view of health promotion.

A literature review showed that long-distance running can be a form of therapy, and people also run to improve their mental health (16). Parkrun has had a positive impact on mental health. Studies have indicated that depression, tension, isolation and anger decreased, while participants' self-esteem, mood and stress levels improved (44, 45). According to Stevinson et al. (46) happiness and stress reduction were maintained 1 year after starting Parkrun (47). In Australia, well-being improvement was limited to older runners. Women's personal well-being may benefit from Parkrun especially through improved mental health, and men's well-being may be enhanced by their being connected to a community. Grunseit et al. (33) underlined that Parkrun may facilitate social identity and continuation of healthy habits among athletes, and non-demanding, health-enhancing activity among non-athletes. Researchers have shown that, in the early stages, Parkrun participants mainly focus on health benefits, but later social contacts and the opportunity to help and volunteer grow in importance (46). According to Wiltshire and Stevinson (48), Parkrun offers a space for collective bodywork whereby participants simultaneously enact personal body projects while they also experience a sense of being, all of which comes together to ameliorate certain individualizing effects of health “responsibilization.” Growing evidence suggests that social identities may have profound implications for physical activity participation. Previous studies about Parkrun have demonstrated that group identification was significantly associated with greater participation, exercise-specific satisfaction, group cohesion, and life satisfaction. Findings provide real-world evidence of the health-related benefits associated with forming strong social identities in exercise settings (49).

According to our study, the highest-rated motivations for beginner runners were related to health orientation and personal goal achievement, whereas the lowest-rated motivations were related to recognition and competition. Almost 75% of respondents made an independent decision to start running in Parkrun and City Trail. In contrast, a statistically significant (higher) difference was found on the Affiliation scale among those who had been encouraged by other people. The Affiliation scale was especially important for single participants who started running in comparison with married runners and participants who were in a relationship. As for family circumstances, the Recognition scale was of higher importance for people who did not have children. The Life meaning scale proved to be more important for people with secondary education level.

The greatest differences in motivations were recorded for gender and age analyses of beginner runners. While men were more likely to start running due to Competition-related motivations, women tended to do so due to aspects related to Affiliation, Psychological Coping, Life Meaning and Self-esteem, which is in line with previous research (10, 11, 13, 14). As far as age was concerned, statistically significant correlations were obtained for Personal goal achievement, Competition and Recognition. As age increased, the level of motivation on these scales decreased. According to marital status, affiliation dimension showed significant differences between engage people and single beginners, in contrast to the results obtained by León-Guereño et al. (14) who did not find any differences among married, engaged and single, in any of the 9 dimensions of MOMS in amateur marathon runners. On the other hand, recognition dimension was found to be statistically different between beginner runners who had children and those who had not, being this variable interesting for further analysis.

The survey results are optimistic. They showed that nearly 75% of respondents made an independent decision to start running in Parkrun and City Trail, and participation in these events motivated them to lead an active lifestyle at a level of nearly 5 points on a 7-point Likert scale.

The key strength of this study is that it is focused on a sample comprised of inexperienced runners. Other than that, athletes' family context is taken into account, being this another innovative perspective of this research, thus analyzing runners' marital status and whether they have children or not. According to Goodsell et al. (50) athletes' motivations need to be understood beyond psychological aspects, and social factors need to be taken into consideration. However, the obtained results need to be viewed carefully, since the research was carried out within a specific social context and using a cross-sectional design that did not allow for any causal inferences. Another limitation involves the use of an online survey to collect the data. However, online studies have been reported to obtain very similar results to those administered manually with paper and pencil (51, 52). In the future, more characteristics of inexperienced runners should be investigated, such as age-related motivations by gender, athletes' health status, or the number of children in the family. Moverover, in the future beginner children should be checked and add our reference about children (53).

## Conclusions

In conclusion, this study shows that social-demographic variables such as gender, age, education, and marital and family status had an impact on the decision to start running, so these factors should be taken into account when promoting mass sport events aimed at enhancing people's health. Fostering safe running among people who have no previous experience is as important as encouraging people to run. All participants stated that they would like to run a marathon in the future.

## Data Availability Statement

The raw data supporting the conclusions of this article will be made available by the authors, without undue reservation, to any qualified researcher.

## Ethics Statement

Ethical approval was not provided for this study on human participants because as online surveys or questionnaires do not require the completion of a separate participant information sheet or consent form, participation in the survey was deemed to constitute informed consent. Participants were informed about the significance of the study and were kindly requested to provide information. The survey was voluntary, anonymous and confidential. In Poland, anonymous diagnostic surveys do not require approval by a bioethics committee. The survey was forwarded to City Trail and Parkrun participants by the events' organizers. Written informed consent was not provided because Ethical approval for this study and written informed consent from the participants of the study were not required in accordance with local legislation and national guidelines.

## Author Contributions

EM-M contributed conception and design of the study and organized the database. EM-M and ZW performed the statistical analysis and wrote the first draft of the manuscript. EM-M, PL-G, MT-S, and PS-M wrote sections of the manuscript. All authors contributed to manuscript revision, read, and approved the submitted version.

## Conflict of Interest

The authors declare that the research was conducted in the absence of any commercial or financial relationships that could be construed as a potential conflict of interest.
